# Investigation of the safety features of advanced PWR assembly using SiC, Zr, FeCrAl and SS-310 as cladding materials

**DOI:** 10.1038/s41598-021-96954-9

**Published:** 2021-08-31

**Authors:** Sayed. Saeed. Mustafa

**Affiliations:** Faculty of Science, Zagazig University, Zagazig, Egypt

**Keywords:** Theoretical nuclear physics, Nuclear physics

## Abstract

In this work, SiC (Silicon carbide), FeCrAl (ferritic), SS-310 (stainless steel 310) and Zirconium are simulated by MCNPX (Monte Carlo N‐Particle eXtended) code as cladding materials in advanced PWR (Pressurized Water Reactor) assembly. A number of reactor safety parameters are evaluated for the candidate cladding materials as reactivity, cycle length, radial power distribution of fuel pellet, reactivity coefficients, spectral hardening, peaking factor, thermal neutron fraction and delayed neutron fraction. The neutron economy presented by Zr and SiC models is analyzed through the burnup calculations on the unit cell and assembly levels. The study also provided the geometric conditions of all cladding materials under consideration in terms of the relation between fuel enrichment and cladding thickness from the viewpoint to achieve the same discharge burnup as the Zircaloy cladding. It was found that the SiC model participated in extending the life cycle by 2.23% compared to Zr. The materials other than SiC largely decreased discharge burnup in comparison with Zircaloy. Furthermore, the claddings with lower capture cross-sections (SiC and Zr) exhibit higher relative fission power at the pellet periphery. The simulation also showed that using SiC with a thickness of 571.15 μm and 4.83% U-235 can satisfy the EOL irradiation value as Zr. For reactivity coefficient, the higher absorbing materials (SS-310 and FeCrAl) exhibit more negative FTCs, MTCs and VRCs at the BOL But, at the intermediate stages of burnup Zr and SiC have a strong trend of negative reactivity coefficients. Finally, the delayed neutron fraction of SiC and Zr models is the highest among all the four models.

## Introduction

Because of the inherent resistance of Zirconium alloys to a variety of environmental conditions, they have been used effectively as cladding materials in light water reactors^1^. Although these alloys have many advantages as the excellent neutron economy and the small capture cross sections, their resistance to oxidation is reduced when subjected to high temperatures during the reactor operation. Consequently, this will result in an increase of hydrogen absorption that affects the microstructure of the material beside it loses ductility as the time proceeds^2^. After 2011, many investigations are carried on other cladding materials that can displace Zirconium^3^. These investigations included a lot of studies on mechanical, irradiation and corrosion characteristics including reactions with water, and interaction behavior with fuel^4^. In this study, FeCrAl and SS-310 are one of the alternative cladding materials used in the advanced PWR assembly. The capture cross section of these materials cause a reduction in the neutron economy in the reactor core.

Zirconium-based alloys under normal operating conditions in the reactor core naturally undergo corrosion process, in which the zirconium dioxide (ZrO_2_) is formed on the metal surface. This corrosion is accelerated when the alloy is subjected to steam with very high temperature, for example, during a Loss of Coolant Accident (LOCA). The excess of hydrogen absorption beyond the limit of solubility leads to the formation of hydrides, which is the main consequence of the ductility loss. This ductility loss is responsible for the weakness of Zirconium based alloys^5,6^.

There is a need for developing new cladding materials that can provide better efficiency in the conversion of energy in LWRs and that are capable of reducing the possible damages in case of accidents. Silicon carbide compounds have many advantages, such as excellent high-temperature properties, good corrosion resistance, low neutron absorption cross-section and no absorption peak of hydrogen during normal operation, and a considerable life increase when the fuel is irradiated at high-burnup values^7^. On the other hand, Usage of SiC materials as a fuel cladding is accompanied by some undesirable properties as low thermal conductivity of irradiated SiC^8,9^ and large stresses that could result in loss of hermeticity expected from these structures^10^. However, the high oxidation resistance^11^ and strength of SiC-based materials in high-temperature steam environments make these materials favorable in the study of ATF (accident tolerant Fuel) concepts.

The SS-310 type contains high chromium and nickel contents and exhibits high temperature steam oxidation resistance by forming a protective Cr_2_O3 scale under these conditions^12^. The ferritic alloy, FeCrAl, contains aluminum which form a protective layer of Al_2_O_3_ under high temperature steam oxidation conditions^12^. The protective layer formed on the periphery of the cladding tube are oxidized slowly under high temperature steam environments and are thus considered safer under accident circumstances. The high Ni, Cr and Fe content in SS-310 cladding is responsible for its high neutron capture properties, which Campaign to develop novel fuel and cladding concepts to replace the current Zr alloy-UO_2_ fuel system. The interaction of Zr with reactor coolant as water is one of the disadvantages of Zr-based claddings that results in a reduction of the material's ductility under normal operating conditions^13^.

The present work investigated the neutronic performance of SiC, FeCrAl, SS-310 and zirconium alloy using burnup calculations on the unit cell and assembly levels. Isotope depletion analyses, capture cross sections, spectral and spatial self-shielding analysis, plutonium radial profile and reactivity coefficients are studied and analyzed for each cladding material. Furthermore, delayed neutron fraction is also evaluated for the candidate claddings. Moreover, MCNPX simulation allowed us to determine the enrichments of UO_2_ and the thicknesses of SiC, FeCrAl and SS-310 necessary for achieving the EOL burnup of Zr case through an assembly geometry optimization. The neutronic and burnup analysis were made by MCNPX2.7 code.

The results of this paper shows that the calculated safety parameters of SiC cladding model are similar to those of conventional Zr cladding model. This is attributed to the low absorption cross section of both materials and this in turn improves safety and economy.

Several researchers have studied the neutronic performance of the cladding materials under consideration. Allam et al.^14^ have calculated reactivity, radial power distribution and spectral hardening for small modular reactor assembly using micro-heterogeneous duplex fuel. They performed their calculations by WIMS-10 Lattice physics code (deterministic method) using nuclear data from the JEF 2.2 database available from the IAEA. Naceur and Marleau^15^ compared the neutronic performance of (SS-304 and SS-310), (FeCrAl and APTAM) and SiC with Zirconium alloy using burnup calculations, isotope depletion analyses, absorption rate ratios, spectral and spatial self-shielding analysis, plutonium radial profile and reactivity perturbations. Their calculations are based on the 3D lattice transport-theory code DRAGON5 which is part of the Industry Standard Tool-set (IST) of Codes for CANDU reactor core design and analysis. George et al.^11^ used SCALE 6.1 code with cross section library, ENDF/B-VII.0 to analyze the neutronics associated with each cladding material as burnup penalty, changes in reactivity coefficients, and spectral variations. But in the present work, the author carried out the neutronic and depletion calculations with MCNPX code which depends on the Monte Carlo method using cross section library ENDF/B-VII.1. This means that there is a difference in the methodology and the cross section library used in the present work and other published works. All the previously mentioned works depended on a deterministic method or coupling between a Monte Carlo method with a deterministic one. Therefore, one can say that the present work is considered the first one that used MCNPX code for simulating the effectiveness of these cladding materials in the advanced PWR assembly.

## The cladding materials under investigation

Table 1 depicts the cladding materials with their isotopic compositions (in weight percent). In our study, the baseline zirconium alloy (Zircaloy-II) is compared with three different candidates: silicon carbide (SiC), ferritic iron chromium aluminum alloy (FeCrAl) and austenitic type (SS-310). These claddings were considered because of their sufficient oxidation resistance at a significantly higher melting point than for Zircaloy.

**Table 1:** Candidate cladding materials with their elemental composition^15^.

Material	Fe	Cr	Al	Zr	Ni	Sn	Mn	Mo	Si	C
SiC									70.08	29.92
Zircaloy	0.15	0.1		98.36		1.49				
FeCrAl	75	20	5							
SS-310	55.55	25.22			19.51		1.9	0.122		

Table 2 shows the microscopic thermal capture cross sections of the cladding materials under investigation. Materials with higher absorption properties as FeCrAl and SS-310 are expected to reduce neutron economy in the reactor core as more neutrons are absorbed in these materials before being captured in the UO_2_ fuel region. Although the absorption properties of a given material are dependent on neutron energy, average thermal cross sections are representative of neutron energies after they have been slowed down by the moderator, which is the energy range where most fission reactions occur in a PWR.

**Table 2:** Microscopic thermal neutron absorption cross section for various cladding materials^16^.

Material	Density g/cm^3^	Absorption cross section (barn)
SiC	2.58	0.086
Zircaloy	6.56	0.2
FeCrAl	7.1	2.43
SS-310	8.03	3.21

## The physical model under investigation

**Figure 1:**
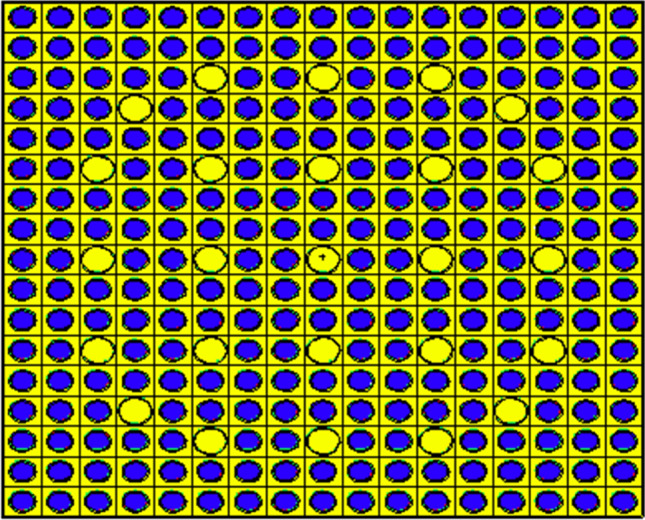
Advanced Westinghouse PWR assembly of reference case modeled by MCNPX code.

The two-dimensional pin cell and assembly models of the candidate claddings are carried out by MCNPX2.7 code. Figure 1 depicts advanced 17 × 17 PWR assembly which is taken the reference case (Fuel–Zirconium combination). The assembly contains 246 normal fuel pins and 25 guide tubes. The boron concentration in that assembly is 0 ppm. The specifications of the fuel assembly are indicated in Table 3. The UO_2_ fuel rods are enriched to 4.9%. Refractive boundary conditions are taken into consideration during the neutronic and burnup calculations. The simulation of pin cell and assembly designs is conducted at constant fuel pitch (1.326 cm) and pellet-cladding gap (0.008255 cm). A comparison is held between the four candidate cladding materials from the point of view of reactivity at constant clad thickness 0.05715 cm.

**Table 3:** The parameters and dimensions of advanced PWR assembly^17–19^.

Fuel assembly rod array	17 × 17
Number of guide thimbles	24
Number of instrumentation thimbles	1
Pin-to-pin pitch	1.326 cm
Number of fuel rods per assembly	264
Assembly fuel height	365.76 cm
Fuel pellet radius	0.409575 cm
Gap thickness	0.008255
Fuel enrichment	4.9%
Cladding outer radius	0.474980 cm
Cladding thickness	0.05715 cm
Cladding inner radius of guide tube	0.5624 cm
Cladding outer radius of guide tube	0.6032 cm
Fuel density	UO2: 10.468 (95.5% theoretical density)
Specific power density for reference UO2-Zr	38.33 Watt/gram
Coolant density	0.7119 g/cm^3^

**Figure 2:**
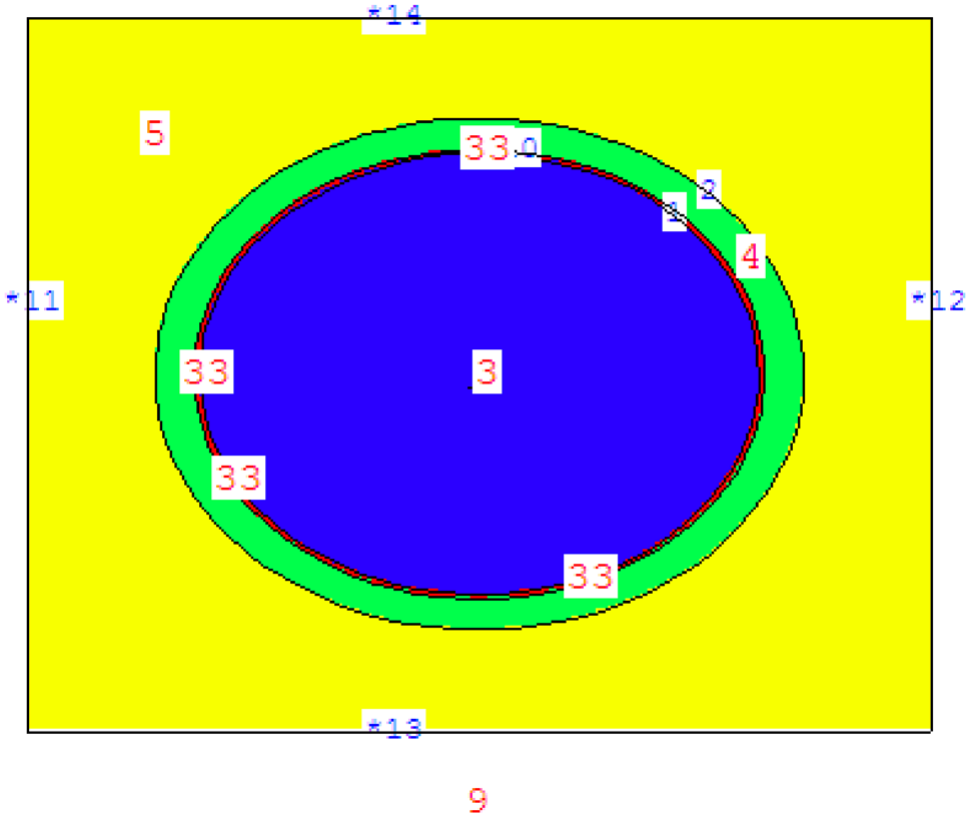
Pin cell geometry of reference case modeled by MCNPX code.

**Figure 3:**
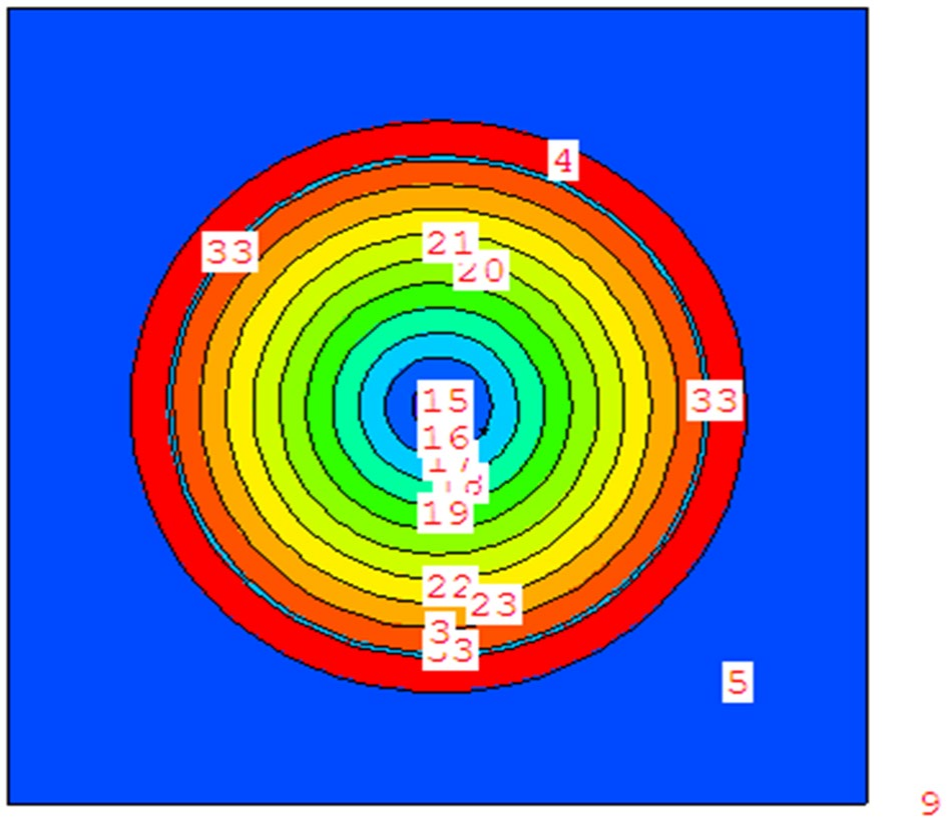
Radial pin cell geometry with 10 equal-volume concentric rings modeled by MCNPX code.

Figure 2 provides the geometry of pin cell which consists of fuel, gap, clad and moderator. The temperatures of fuel, clad and moderator are 900 K, 600 K and 600 K respectively. For studying the self-shielding effect, the fuel pellet geometry is divided into 10 equal concentric rings as in Fig. 3.

## Method of calculation

MCNPX (MCNP eXtended) is a Fortran90 (F90) Monte Carlo radiation transport computer code that transports nearly all particles at nearly all energies. All the neutronic and burnup calculations in this research are conducted by MCNPX code. This code is capable of modeling complex 3D geometries and utilizes extensive point-wise cross-section data library on a continuous energy spectrum. The cross section data library used in the calculations is the updated library, ENDF/B-VII.1. This data library provides the necessary probability distributions for simulating particle interactions through use of random number sampling^20^.

## Results and discussions

### Depletion analysis for unit cell models

In order to analyze the effect of different cladding materials on the assembly neutronics, a PWR pin cell geometry shown in Fig. 2 was modeled with MCNPX code. The pin cell is composed of 4.9% enriched UO_2_ fuel pellet with density of 10.468 g/cm^3^. There is Helium gap of density 0.001625 g/cm^3^ between fuel pellet and cladding. Water is used as a coolant and moderator. The pin cell with Zircaloy clad was selected as reference case. The burnup calculations are carried out at a constant power of 38.33 MW per metric ton of Uranium (MTU) for a discharge burnup value 60 GWd/ton which corresponds to 1565 effective full power days. Four models were simulated by MCNPX code. These models have the same parameters and dimensions except for the cladding material type. The cladding materials are SiC, Zircaloy, FeCrAl and SS-310. The composition of clads used for calculations is summarized in Table 1.

**Figure 4:**
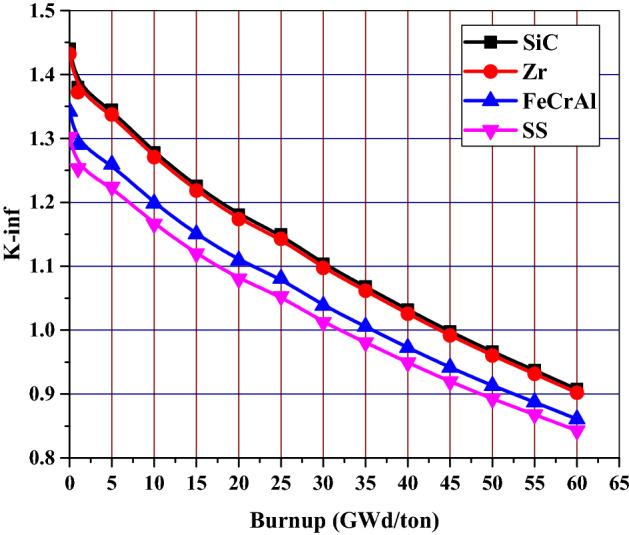
The evolution of K-infinity with burnup for the cladding materials under investigation.

Figure 4 shows the variation of K-infinity (infinite multiplication factor) with time for 4 different cladding materials. When compared with the reference case, the highest decrease in k-infinity occurs for 310 stainless-steel cladding case. The increase in plutonium inventory on the unit cell level of this proposed cladding design (SS-310) is the cause of this K-infinity reduction. Only increase in k-infinity occurs for SiC cladding case due to reduction in neutron absorption, since the absorption cross section of SiC is 0.086 barn (10^–24^ cm^2^) compared to 0.20 barn of Zircaloy.

**Figure 5:**
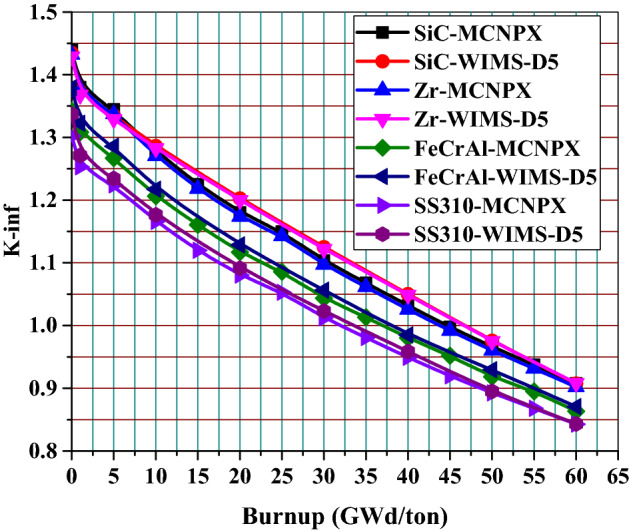
Comparison of K-inf results for MCNPX and WIMS-D5 for the 4 candidate claddings.

For validation, the infinite multiplication factor of the four pin cell models is obtained firstly by MCNPX code using ENDF/B-VII.1 nuclear data library. Then the same models are simulated by WIMS-d5 code using WLUP-69.bib library. The K-inf results of the two codes are compared with each other. The results in Fig. 5 show that there is a good agreement between the two code results for all the candidate claddings.

According to the analysis of Table 4, SiC cladding provides the highest end of life (EOL) burnup among the other cladding materials. On the other hand, SS-310 cladding has the lowest EOL burnup. This means that that length of the fuel cycle decreases when Zircaloy cladding is replaced with either 310 stainless-steel (26.5% reduction) or FeCrAl (21.3% reduction), but increases when it is replaced by SiC (2.23% increase).

**Table 4:** Calculated discharge burnup of proposed models.

Cladding material	Discharge burnup (GWd/ton)	Effective full power days
SiC	44.666707	1165.316
Zircaloy	43.666699	1139.228
FeCrAl	36.000004	939.212
SS-310	32.083330	837.029

**Figure 6:**
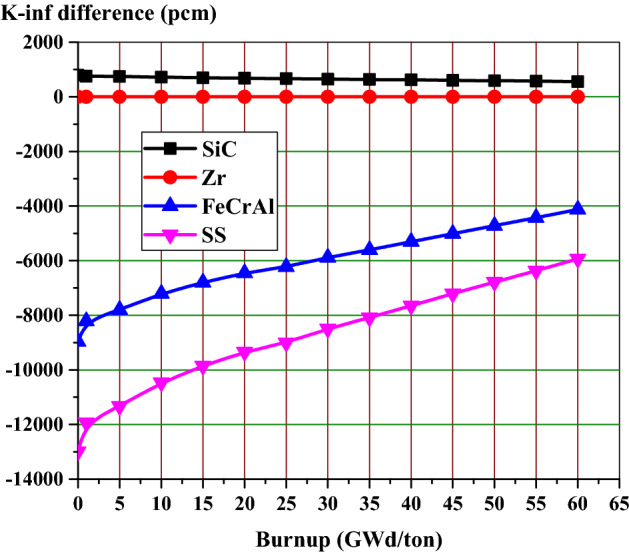
K-infinity difference from Zircaloy clad fuel versus burnup for various cladding materials.

The remarkable drop in K-infinity values found for SS-310 and FeCrAl corresponds to a significant reduction in operational cycle length. As depicted in Fig. 6, the absolute value of k-infinity difference for those two cladding materials decreases throughout the cycle. This is attributed to the increased plutonium content throughout the cycle in the presence of a harder neutron spectrum. The reactivity penalty at 60 GWd/ton for SS-310 were found to be nearly 5.933% k-infinity difference, while the ferritic alloy (FeCrAl) was slightly less negative at 4.123%. The less absorbing SiC cladding produced a positive 0.555% k-infinity difference at end of life (EOL).

This part concludes that the replacement of Zircaloy cladding with SiC will provide K-inf and penalty benefits. Meanwhile, the penalty associated with the other candidate claddings (SS-310 and FeCrAl) necessarily shortens the core life, which is undesirable to our design objective of achieving a long-life advanced PWR core.

### Analysis of self-shielding on the radial power distribution

**Figure 7:**
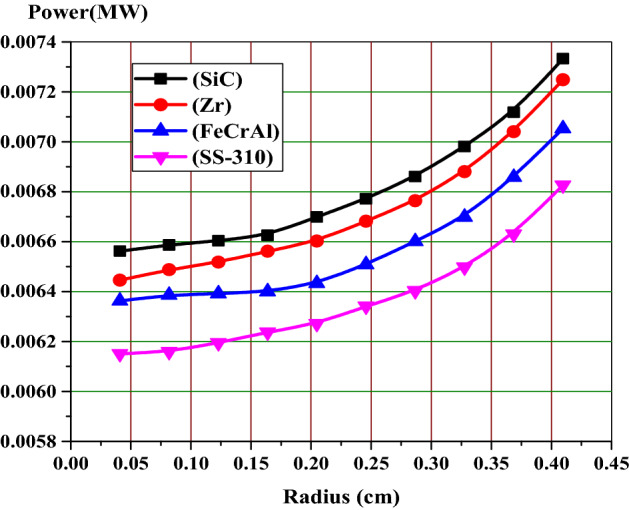
Relative radial fission power distributions at BOL.

**Figure 8:**
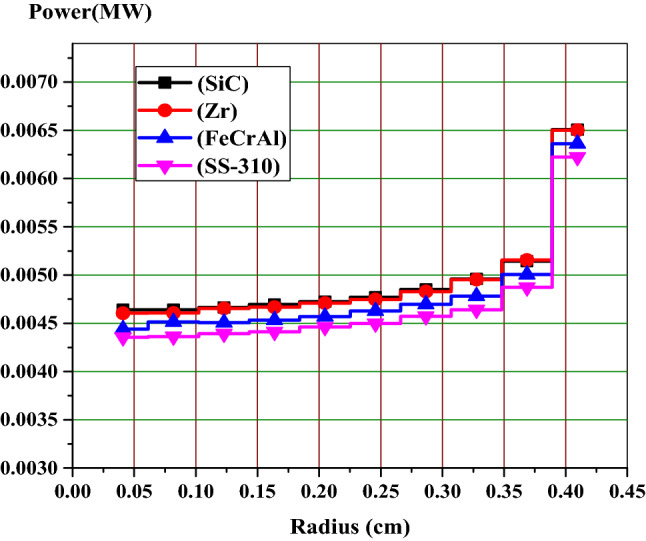
Relative radial fission power distributions at the middle of life (MOL).

**Figure 9:**
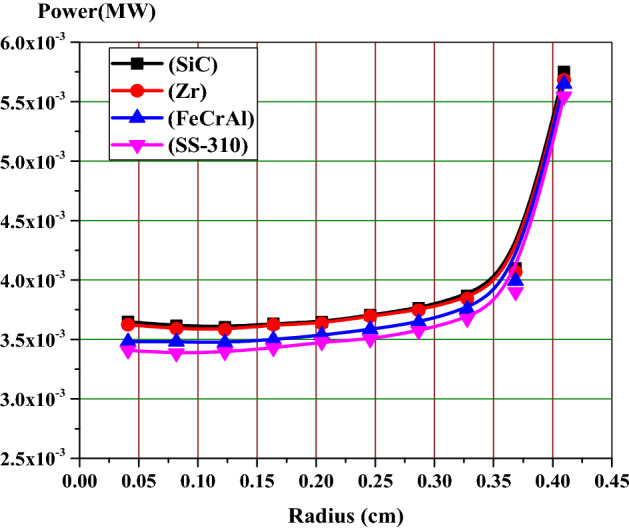
Relative radial fission power distributions at the end of life (EOL).

The self-shielding effect of the fuel rod causes a non-uniform radial power distribution. This is caused by the strong absorption of U-238 near the surface of the fuel rod. This in turn effects on the plutonium production and burnup at this position. Therefore, it is important to analyze the rim effect when considering the four cladding materials under investigation. The fuel region is divided into 10 rings of equal volumes, while the gap and cladding are located outside with their same dimensions. Figures 7, 8 and 9 illustrate the fission power in MW across the fuel pellet at BOL, MOL and end-of-life (EOL) for all candidate claddings for UO_2_ fuel (4.9% U-235). All figures show that the power is largest in the region next to cladding material (at fuel rod surface) and smallest at the center of the fuel rod due to spatial self-shielding as stated before. Because of the significant differences between the capture cross sections of different clads, the fission power values are higher for SiC model and lower for SS-310.

**Figure 10:**
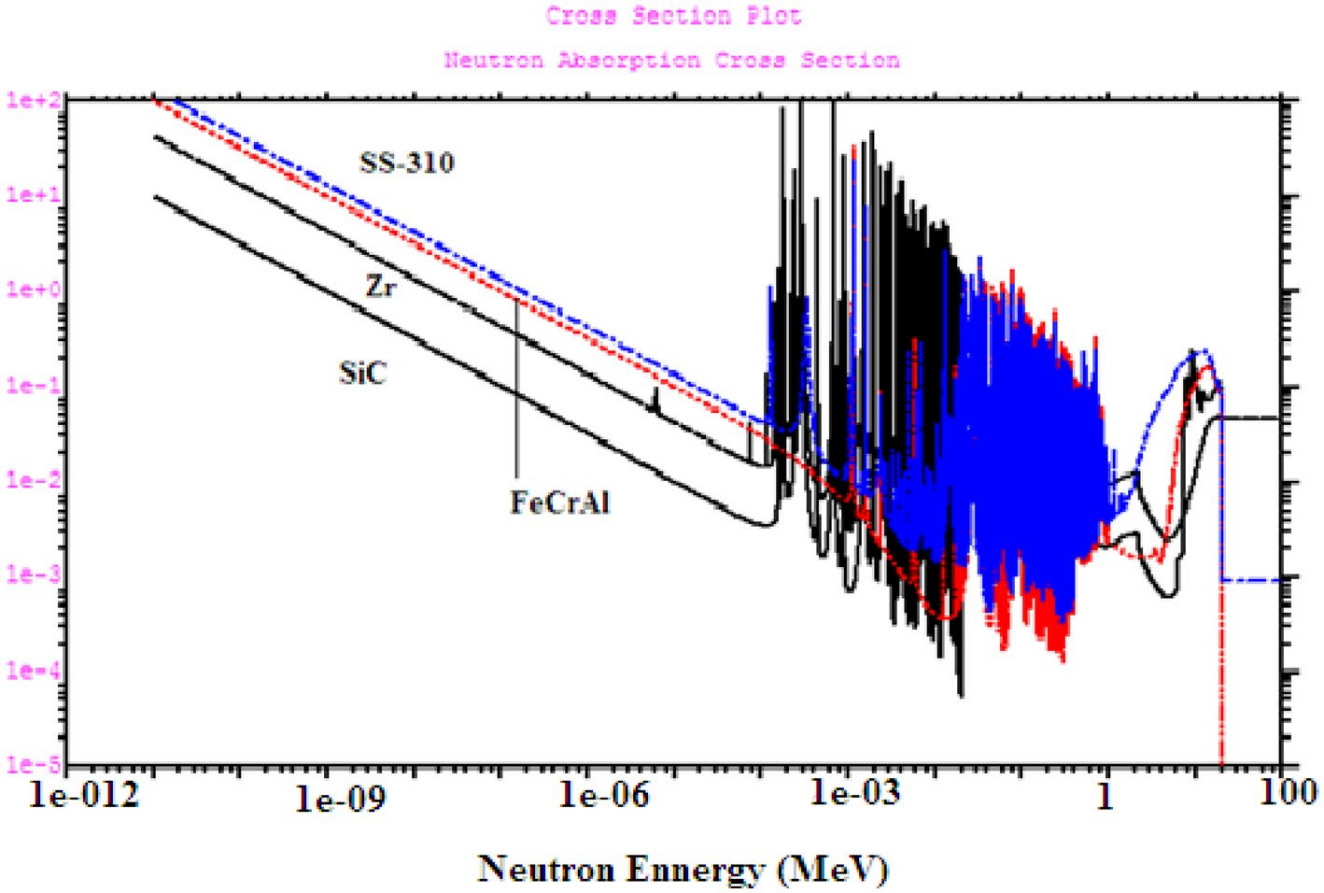
Absorption cross section in (barn) versus energy (Mev) for 4 clads by MCNPX.

Figure 10 compares the microscopic capture cross sections of the 4 cladding materials. As one can see, in the thermal region (E < 0.625 eV), the capture cross sections of FeCrAl and SS-310 are higher than those of Zr and SiC. The high absorption cross section of both FeCrAl and SS-310 is mainly due to the fact that these materials contain at least 52% iron, a material that has an absorption cross section in the thermal groups of the order of 10 barns. Owing to the capture cross section of nickel-59, (SS-310) has the highest thermal neutron absorption among the candidate claddings. Nuclei having magic numbers of neutrons (^90^Zr_40_) or a doubly semi-magic number (^28^Si_14)_ are characterized by their low tendency in capturing neutrons.

For Zr and SiC claddings in Fig. 10, the graphs are linear and decreasing until about 10^–6^ MeV to Zircaloy and 0.01 MeV to SiC. It is clear that the SiC has a neutron absorption cross section lower than the Zircaloy, explaining the higher neutron multiplication for the SiC compared to Zircaloy.

The serrated peaks in Fig. 10 refer to the high absorption cross section of the candidate claddings for the epithermal neutrons (10 eV to < 1 MeV). As observed in the figure, the average epithermal absorption cross section of Zr is higher than that of SiC in this energy range. As a result, the epithermal flux of SiC is expected to be higher than that of Zr. On this basis, SS-310 has a lower epithermal flux compared to FeCrAl.

For fast neutron region at about 1.22 MeV, there are fast flux peaks which corresponds to low absorption cross sections for the candidate materials at this high energy range. These fast flux peaks will be highest and lowest for SS-310 and SiC respectively as in Fig. 14. This concludes that the hardening of the spectrum is found at the BOL as the thermal peaks at 0.1 eV decrease (from SiC till SS-310) and the fast peaks at 1.22 MeV increase (from SiC till SS-310). This phenomenon is due to the high thermal and epithermal absorption cross section of SS-310 and FeCrAl that contributes to decreasing the thermal flux and increasing the fast flux.

**Figure 11:**
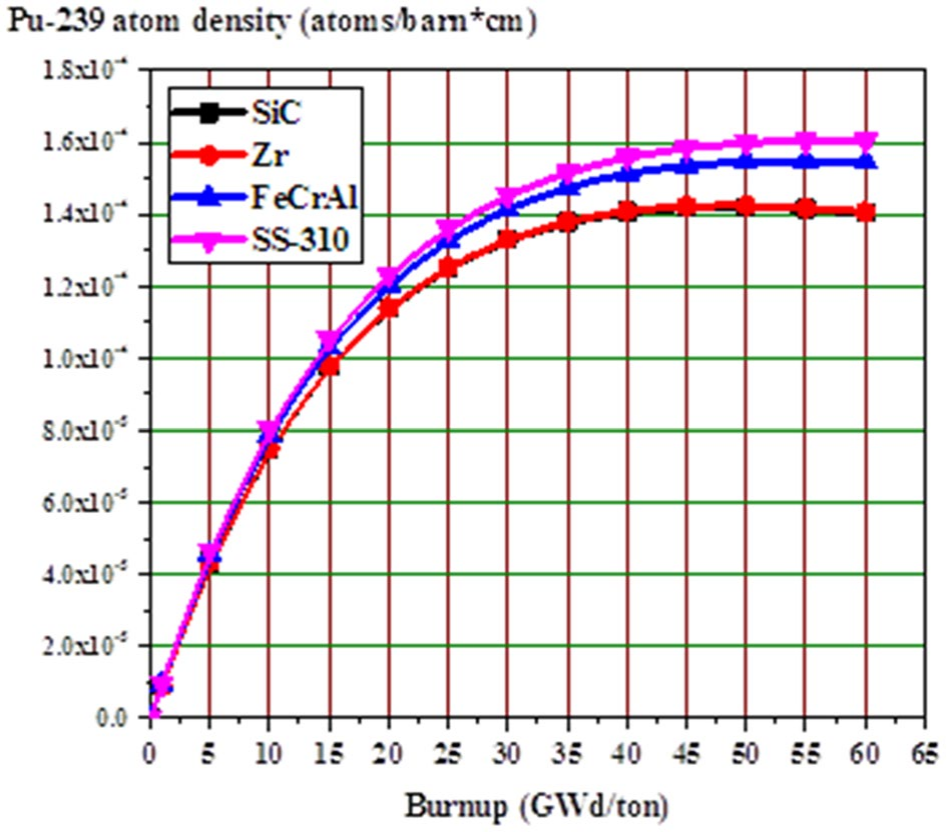
Pu-239 atom density versus burnup for all candidate claddings.

The fission power behavior obtained by the self-shielding effect can be explained by Pu-239 atom density at MOL and EOL as a function of pellet radius. Because of the large contribution of Pu-239 to fission after U-235, Pu-239 is chosen for this analysis. Figure 11 shows the buildup of Pu-239 in the fuel pellets with burnup. The Pu-239 concentration increases differently with burnup stages for the 4 different claddings. As expected, the build-up of Pu-239 is higher in SS-310 model and lower in SiC model.

The increased concentration of Pu-239 with burnup (from MOL to EOL) is owing to the spatial self-shielding of neutrons, where thermalized neutrons are captured in the outer region and thus blocked from the center of the fuel. The higher Pu-239 production at the pellet periphery results in very high burnup in the rim region and greater local fission gas release, which is responsible for the formation of porosity in this region.

**Figure 12:**
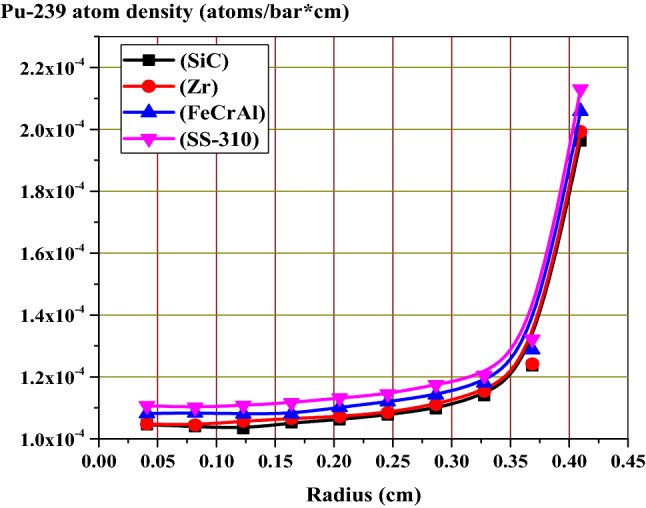
Radial distributions of Pu-239 atom density at the middle of life (MOL).

**Figure 13:**
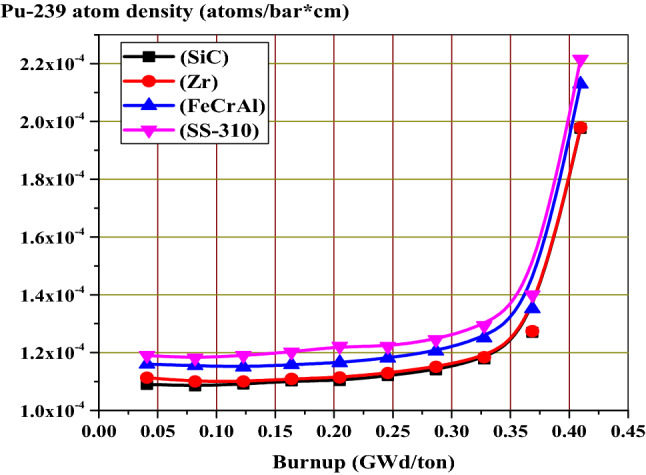
Radial distributions of Pu-239 atom density at the middle of life (MOL).

In Figs. 12 and 13, the buildup of Pu-239 on the periphery of the fuel pellet is significantly greater than in the center due to strong epithermal U-238 resonance absorption at this location. Furthermore, the MOL and EOL Pu-239 inventories are higher for FeCrAl and SS-310 with higher thermal capture cross-sections due to spectral hardening, unlike SiC and Zr. FeCrAl and SS-310 claddings absorb more thermal neutrons than Zr or SiC cladding, thereby decreasing the fast neutron in the later. This in turn increases the resonance capture in U-238, hence more Pu-239 breeding is observed throughout the cycle for the Fe- and steel-based cladding fuels.

### U-235 enrichment and cladding thickness matching cycle length requirements

In this section, the necessary enrichment and cladding thickness to match the fuel cycle length of the reference case (Zirconium model) were calculated for the candidate claddings. For U-235 enrichment, many trials are conducted by MCNPX code for reaching the suitable enrichment that achieve the same EOL burnup value of Zirconium case (43.66 GWd/ton). The cladding thicknesses for the SiC and Zircaloy cases were kept at 571.5 Μm, which represents a typical thickness for a Westinghouse PWR fuel rod. Meanwhile, the thickness of FeCrAl and SS-310 has to be reduced to 200 Μm. The fuel radius (0.409575 cm), fuel pitch (1.326) and Helium gap thickness were kept constant in all simulations.

The results in Table 5 reveal that SS-310 requires highest U-235 enrichment (5.22%) at the same thickness of FeCrAl (200 μm). This is expected due to the high capture cross section of SS-310 compared to FeCrAl. For SiC and Zr, SiC needs lower enrichment (4.83%) at the same thickness of Zirconium (571.5 μm). This is attributed to the high absorption cross section of Zr compared to SiC. The second part of this section investigated the suitable thickness of SS-310, FeCrAl and SiC that can achieve the EOL burnup value as in Zirconium case. The simulation was performed at constant enrichment, fuel radius, fuel pitch and helium gap but the cladding thickness was varied according to its type.

**Table 5:** U-235 enrichment required for achieving the same EOL burnup of Zirconium model.

End of burnup value (GWd/ton)	Cladding material	Enrichment (%)	Clad radius	Required thickness (μm)
43.66	SiC	4.83	0.466725	571.5
43.66	Zirconium	4.90	0.466725	571.5
43.66	FeCrAl	5.06	0.429575	200
43.66	SS-310	5.22	0.429575	200

As mentioned previously, SS-310 and FeCrAl contain elements with higher neutron absorption than Zircaloy. Therefore, a great heavy metal loading is required to achieve the EOL burnup value of Zircaloy at the constancy of fuel enrichment. This is conducted by using a thinner cladding while maintaining a constant fuel pitch. This means that the increased levels of U-235 enrichment via reducing the cladding thickness also could serve in obtaining the cycle length achieved by Zircaloy. In other words, the required thickness for SiC and SS-310 for obtaining the same cycle length of Zirconium is 770 μm and 105 μm respectively as in Table 6.

**Table 6:** Cladding thickness required for achieving the same EOL burnup of Zirconium model.

End of burnup value (GWd/ton)	Cladding material	Enrichment (%)	Clad radius	Required thickness (μm)
43.66	SiC	4.90	0.486575	770
43.66	Zirconium	4.90	0.466725	571.5
43.66	FeCrAl	4.90	0.424075	145
43.66	SS-310	4.90	0.420075	105

### Spectral hardening in the assembly model

**Figure 14:**
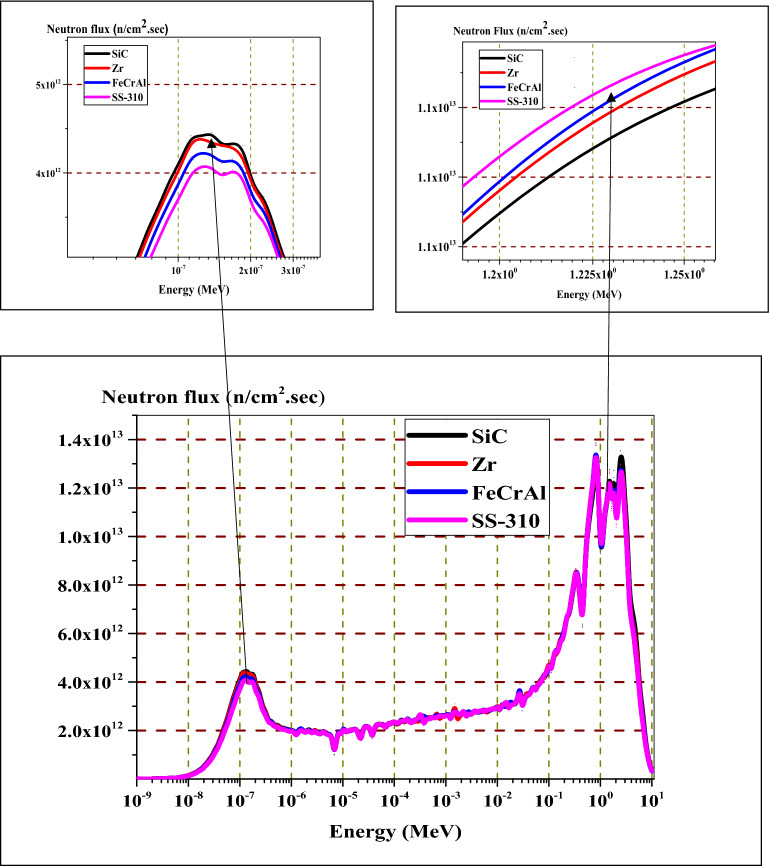
Neutron flux spectrum at beginning of life for the 4 candidate claddings by MCNPX code.

Spectral hardening was analyzed for the SS-310, FeCrAl, Zircaloy and SiC cladding materials under consideration. For this study, the neutron flux in (n/cm^2^ sec) was calculated by MCNPX code across a homogeneous cell of fuel, cladding, and moderator. Then, this flux was plotted against the neutron energy in MeV (Fig. 14). The cross section library used by MCNPX is ENDF/B-VII.1. Analysis of the thermal peaks at 0.1 eV indicates that SS-310 and FeCeAl presents a lower thermal flux values at the beginning of life. This is because both of the two materials absorb thermal neutrons and this results in increasing the fraction of fast neutrons. Thus, the thermal peaks are lower for SS-310 and FeCeAl compared to those of Zircaloy and SiC.

In the same time, the fast peaks at 1.225 MeV are higher for SS-310 and lower for Zircaloy and SiC. The combination between the two effects results in a hardening of the spectrum. This is attributed to the depletion of overall fissile material and, in turn, an increased accumulation of fission products and actinides in the system. This part concludes that SiC cladding is the least absorbing material among the four claddings, consequently it has the highest inventory of thermal neutrons .This results in a softening of neutron spectrum which improves neutron economy and discharge burnup. In contrast, FeCrAl and SS-310 contain more absorbing materials, more thermal neutrons are absorbed in these claddings, resulting in an increase in the fast neutrons fraction in the system compared to the Zr and SiC cladding cases.

### Fuel temperature coefficient

It is important to evaluate the effect of SiC, FeCrAl and SS-310 claddings on the fuel temperature coefficient to compare their feedback response with that of the reference Zr cladding. For maintaining the safety standards of advanced PWRs, it is necessary that each cladding material produces negative fuel temperature coefficients especially at the earlier stages of fuel irradiation. For estimating the FTC, the temperature of moderator and clad was fixed at 600 K but the fuel temperature is increased from 900 K (operational temperature) to 1200 K i.e., temperature change is 300 K.

**Figure 15:**
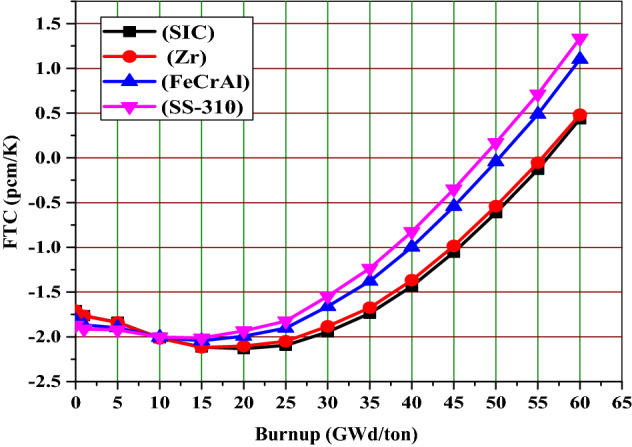
The fuel temperature coefficient as a function of burnup for the 4 claddings.

**Figure 16:**
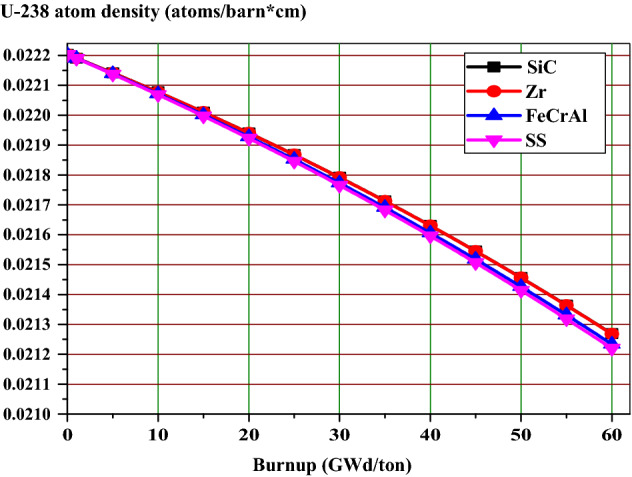
U-238 atom density as a function of burnup for the 4 claddings.

As can be noted in Fig. 15, the FTC values for all cases are less negative at the BOL (0 GWd/ton) and more negative as the burnup increases to about 15 GWd/ton due to the changes in isotopic composition and the increased variety of isotopes present. Table 7 shows that the BOL FTC values are negative for all the candidate claddings. It is also observed that there is slight variation in FTC values owing to the differences of capture cross sections of these materials. The SS-310 and FeCrAl claddings exhibit marginally more negative FTC value compared to SiC and Zr at BOL. This is attributed to the Doppler broadening of fertile absorption. This can be explained via the variation of U-238 concentration with burnup. As shown in Fig. 16, the concentration of U-238 is higher for SiC and Zr compared to FeCrAl and SS-310 at all burnup stages. In other words, For the SiC case, there is a strongly negative feedback coefficient throughout the burnup progress. The SS-310 case has the weakest value. The lower absolute value of FTC is a result of the influence of Pu-239 and Pu-241 and the contribution of the reduced resonance absorption.

**Table 7:** The FTC in pcm/K at BOL for the 4 claddings for a 300 K change in fuel temperature.

Cladding material	FTC (pcm/K)
SiC	− 1.704
Zr	− 1.708
FeCrAl	− 1.82
SS-310	− 1.87

The fuel temperature coefficients of SiC, FeCrAl and SS-310 claddings are negative till 55, 50 and 48 GWd/ton respectively. This means that SiC case contributes more negative FTC values at longer periods and this is a very important safety aspect because the reactor stabilizes as an increase of the fuel temperature. The negative fuel temperature reactivity coefficient is fundamental because in this case occurs the broadening of resonances and an increase in the neutrons absorption, reducing the thermal flux and thus reactivity and temperature.

### Moderator temperature coefficient

Table 8 depicts that the MTC values at BOL are negative for all the cladding materials. The SS-310 and FeCrAl have more negative MTC values than those of Zr and SiC. The slight variations in MTC for the candidate claddings are due to differences in total capture cross section of fuel and cladding. This is indicative of the extent to which resonance capture plays a role, and the resultant impact on MTC. Figure 9 confirms that SS-310 and FeCrAl exhibit resonant behavior in the range 0.01–100 eV and thereby provide lower (more negative) MTC values compared to the Zr and SiC cases. The reason for more negativity of MTC of SS-310 and FeCrAl claddings is the presence of Mo and Ni, and Mo isotopes.

**Table 8:** The MTC in pcm/K at BOL for the 4 claddings for a 300 K change in fuel temperature.

Cladding material	MTC (pcm/K)
SiC	− 7.54
Zr	− 7.85
FeCrAl	− 7.93
SS-310	− 8.20

**Figure 17:**
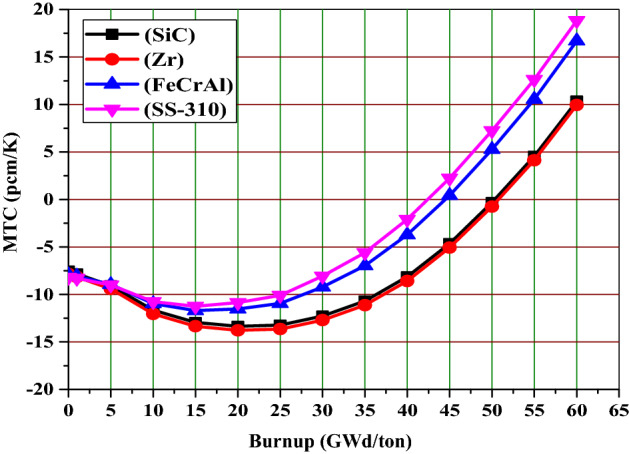
The moderator temperature coefficient as a function of burnup for the 4 claddings.

Figure 17 depicts the moderator temperature coefficient versus burnup for the candidate claddings. This parameter is evaluated when the temperatures of fuel and clad are fixed at 900 K and 600 K respectively. Meanwhile, the moderator temperature is lowered from 600 to 300 K resulting in a temperature change 300 K. Then, using the equation $\partial$ =$\frac{\Delta \rho }{\Delta T}$, the MTC can be calculated in pcm/K. where $\partial$ is the temperature coefficient, $\Delta \rho$ is the reactivity difference, and $\Delta T$ stands for temperature difference which is equal to 300 K.

According to Fig. 17, MCNPX simulation predicts that SiC, FeCrAl and SS-310 show negative MTC till 50, 44 and 42 GWd/ton. There is no a clear difference in the early stage. In the intermediate stages, the Zr case has a more strongly negative coefficient than the other cases. Conversely, the SS-310 case has the weakest negative coefficient.

**Figure 18:**
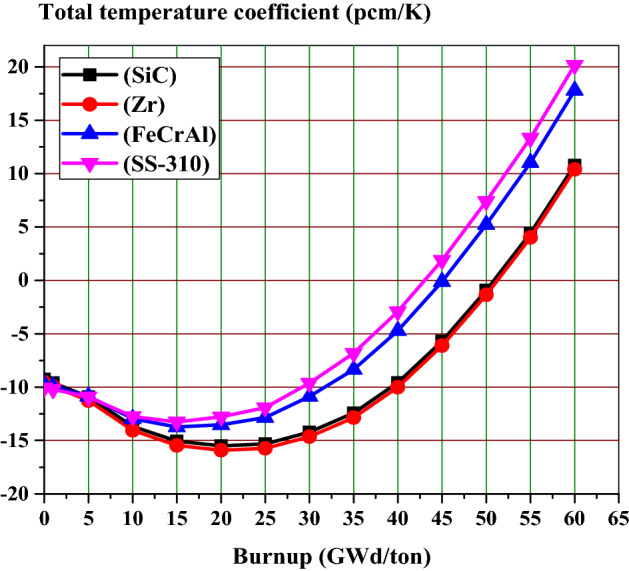
The total temperature coefficient as a function of burnup for the 4 claddings.

The fuel and moderator temperature coefficients are the two most important elements of the reactor feedback coefficient. Figure 18 combines these two temperature coefficients into one total feedback coefficient. Throughout all the burnup stages, the Zr case has the most strongly negative coefficient. The SS-310 and FeCrAl cases have weaker negative feedback coefficients than the Zr case.

To briefly summarize the temperature feedback coefficient results, SS-310 and FeCrAl have the lowest values of fuel and moderator temperature coefficients. The SiC case has more negative FTCs and less negative MTCs compared to Zr case. This results in higher total temperature coefficient values for Zr case compared to SiC case. At the end of life burnup value (60 GWd/ton), the most strongly positive coefficient is observed in the SS-310 and FeCrAl cases.

### Void Reactivity coefficient

For evaluating the void reactivity coefficient, the infinite multiplication factor is calculated at two different void cases. The first one is at 0% void (Density of water = 0.7199 g/cm^3^) and the other is at 40% void (Density of water = 0.4471 g/cm^3^). The void reactivity coefficient (VRC) is defined as the difference in reactivity over the void difference according to the following equation VRC (pcm/%) = $\left(\frac{{\uprho }_{40\mathrm{\%}}- {\uprho }_{0\mathrm{\%}}}{{\mathrm{Void}}_{40\mathrm{\%}}- {\mathrm{Void}}_{0\mathrm{\%}}}\right)*{10}^{5}$ where, ρ_40%_ is the reactivity at 40% void, ${\uprho }_{0\mathrm{\%}}$ is the reactivity at 0% void and VRC is the void reactivity coefficient in units of pcm/%. At the beginning of life as shown in Table 9, The SS-310 and FeCrAl have the highest negative values of VRC among the four cladding materials.

**Table 9:** The VRC in pcm/K at BOL for the 4 claddings.

Cladding material	VRC (pcm/%)
SiC	− 127.46
Zr	− 133.06
FeCrAl	− 135.10
SS-310	− 139.54

**Figure 19:**
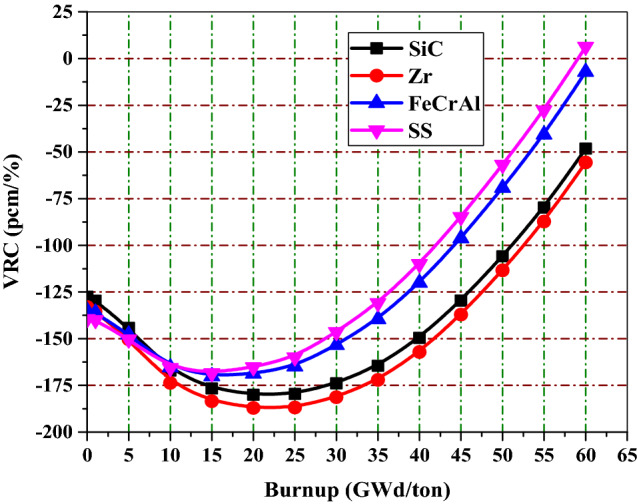
The void reactivity coefficient as a function of burnup for the 4 claddings.

From Fig. 19, the Zr and SiC fuel combination maintain the more negativity of VRC values at all the burnup steps. This guarantees the safe operation of the nuclear reactor.

### Peaking factor

The pin peaking factor is defined as the maximum pin power divided by the average assembly power. It also describes maximum energy generated in the fuel rod on the assembly level. It is used to prevent fuel from reaching a melting point during operation.

**Figure 20:**
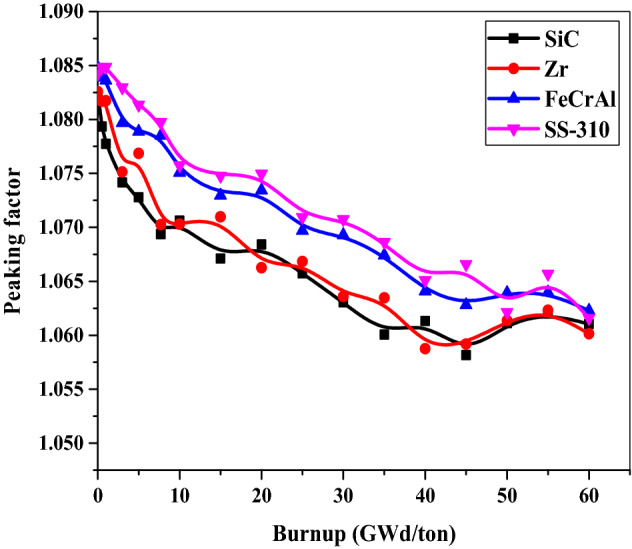
The pin power peaking factor as a function of burnup for the 4 cladding.

Figure 20 illustrates the pin power peaking factor versus burnup stages for the 4 fuel-clad combinations. Zr and SiC claddings have almost the same peaking factor values through the fuel burnup. The peaking factor values for the case of SS-310 and FeCrAl cladding are larger than the other cases (Zr and Sic). Therefore, the use of SS-310 and FeCrAl cladding may lead to smaller shutdown margins which in turn reduces the safety of reactor operation. This means that the use of Zr or SiC are favorable claddings from the side of reducing the peaking factor throughout the burnup stages.

### The thermal neutron fraction

**Figure 21:**
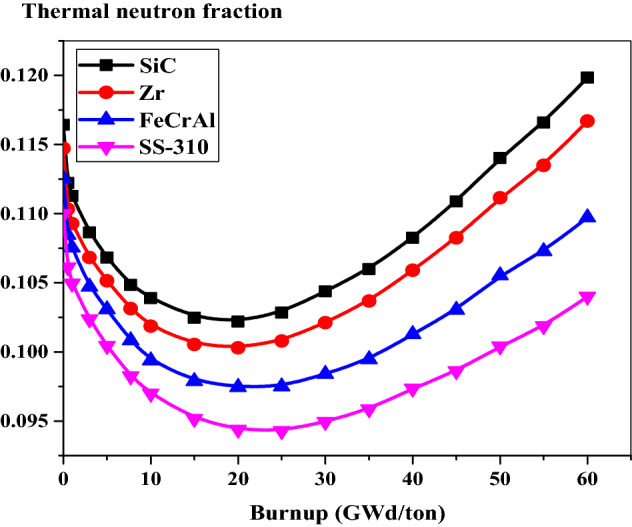
The thermal neutron fraction as a function of burnup for the 4 cladding.

As can be seen in Fig. 21, the thermal neutron fraction decreases with burnup for all cases till 20 GWd/ton. This is a result of the depletion of U-235 as the burnup proceeds. After 20 GWd/ton, the thermal neutron fraction increases with time till 60 GWd/ton. This is attributed to decreasing the macroscopic fission cross sections as the burnup proceeds. This causes increasing the thermal flux with burnup at a constant specific power assumed for each fuel-clad type. It is noticed that the thermal neutron fraction is lower for SS-310. This is because a large fraction of the neutrons will be in the fast and epithermal range and hence captured by U-238. On the other hand, SiC case has the highest values of thermal neutron fraction among the other cases.

### The effect of candidate claddings on the fission products and actinides

**Figure 22:**
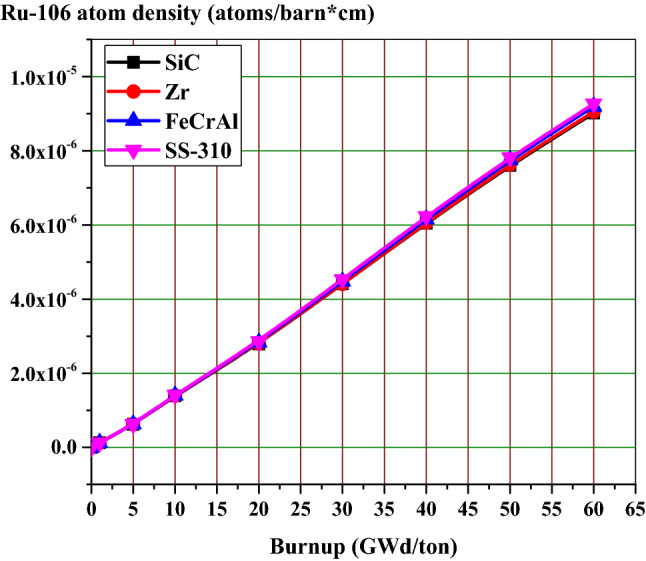
Ru-106 atom density as a function of burnup for the 4 cladding.

**Figure 23:**
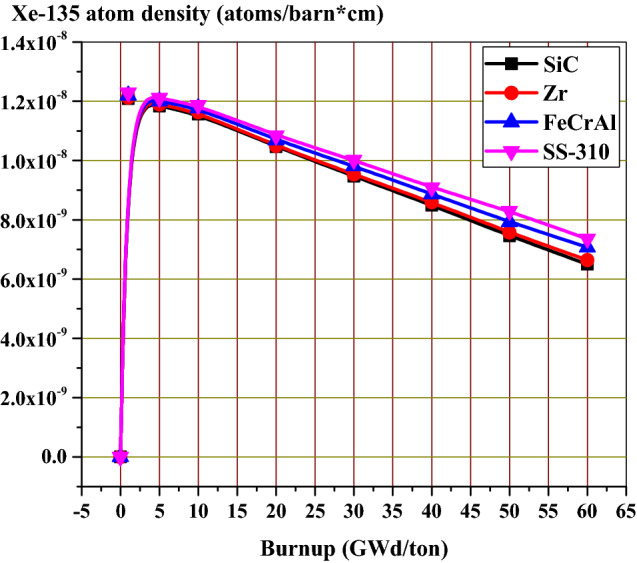
Xe-135 atom density as a function of burnup for the 4 cladding.

As stated before, the calculated normalized flux over fuel, cladding and moderator mixtures can be differentiated in the thermal region. The absorbing materials of high capture cross sections allow fewer thermal neutrons to reach the fuel. For this reason, thermal peaks are arranged (higher for SiC, Zr, FeCrAl then SS-310), while fast peaks may appear superimposed. Lowering the thermal flux in the assembly leads to increasing the fast neutron fraction which in turn causes hardening of neutron flux spectrum. This spectral hardening was found at every depletion step in fuel, cladding and moderator mixtures. Consequently, in the case of spectral hardening, there is less accumulation of actinides and fission products as Ru-106 inventory in Fig. 22 and Xe-135 in Fig. 23.

### Calculation the delayed neutron fraction (${\varvec{\beta}}{\varvec{e}}{\varvec{f}}{\varvec{f}})$ at the BOL for the suggested models

The presence of delayed neutrons plays a significant role in reactor control due to its impact on reactor power change rate. Without delayed neutrons, a reactor would increase in power to such a magnitude and in such a short time period that a significant damage would result. The delayed neutron fraction for each of the 4 suggested models was calculated by using MCNPX code. The calculations are carried out at 10,000 neutrons, 100 skipped cycles and 1000 active cycles resulting in a standard deviation ranging from 0.00018 to 0.00019. It can clear that $\beta eff$ of SiC and Zr models is higher than that of FeCrAl and SS-310 ones. This is because SiC and Zr have low capture cross sections that result in the availability of more fission reactions to occur. Consequently, there will be increasing in the delayed neutron. This ensures the more safety of reactor operation in case of Zr and SiC clads. Table 10 depicts the delayed neutron fraction for the four candidate claddings.

**Table 10:** Delayed neutron fraction for the candidate claddings.

	SiC model	Zr-model	FeCrAl model	SS-310 model
K-eff	1.43893 $\pm$ 0.00019	1.41187 $\pm$ 0.00019	1.36419 $\pm$ 0.00019	1.3229 $\pm$ 0.00018
K-prompt	1.42912 $\pm$ 0.00019	1.40177 $\pm$ 0.00019	1.35503 $\pm$ 0.00019	1.3142 $\pm$ 0.00018
$\beta eff$	0.00682	0.00715	0.00671	0.00657

## Conclusions

Neutronic physical calculations are carried on 17 × 17 advance PWR fuel assembly cladded with different materials. These materials are SiC, Zr, FeCrAl and SS-310. The study showed that such cladding materials have an impact on some important safety parameters. Among these parameters are reactivity, cycle length, radial power distribution, spectral hardening, thermal flux ratio, moderator temperature coefficient, fuel temperature coefficient, void reactivity coefficient, peaking factor and the variation of fission product and actinides as Ru-106 and Xe-135. All the calculations are performed using MCNPX2.7 code. The conclusion of this work can be summarized as following.

1. The values of k-inf using SiC are always higher than those of Zr, FeCrAl and SS-310 at all irradiation values because the cross-section of thermal neutron absorption for SiC cladding is smaller than that of the other claddings.
2. The excess reactivity of the assembly using SiC cladding is the highest among all the other claddings so the operating cycle length is longer.
3. Claddings with lower capture cross-sections as SiC and Zr presents higher fission power in the outer region of the fuel pellet.
4. The SiC cladding exhibits a softer spectrum compared to Zr, FeCrAl and SS-310. This spectrum softening improves neutron economy and thus the EOL burnup.
5. The production of Pu-239 is higher in case of SS-310 and FeCrAl owing to the hardening of neutron flux.
6. Using SiC with thickness 571.15 μm requires the lowest enrichment (4.83% U-235) to reach the EOL burnup value of Zr.
7. Using SS-310 with thickness 200 μm requires the highest enrichment (5.22% U-235) to obtain the same k-inf value at the EOC as Zr.
8. At constant enrichment (4.9% U-235), SiC and SS-310 require thicknesses 770 μm and 105 μm respectively for achieve the same EOL irradiation value of Zr case.
9. At the BOL, the Mo-containing claddings (APMT and 310SS) exhibit more negative MTC than the reference cladding, which is detrimental to shutdown margin but at the intermediate stages, Zr and SiC have more negative MTCs.
10. The SS-310 and FeCrAl claddings exhibit marginally more negative FTC values compared to SiC and Zr at BOL. The most strongly positive FTC is observed in the SS-310 and FeCrAl cases.
11. Less accumulation of actinides and fission products as Ru-106 and Xe-135 are obtained in case of SS-310 and
12. The delayed neutron fraction of SiC and Zr models is the highest among all the four models

This study recommends that silicon carbide (SiC) and Zirconium (Zr) are the best materials used as cladding materials in PWR cores. These materials contribute to improving critical reactor safety parameters owing to their low absorption cross sections compared to the other cladding materials as SS-310 and FeCrAl. From a neutronic investigation point of view, these materials offer a number of advantages over the others. Among these advantages are prolonging the fuel cycle, more neutron economy in the core, more negativity of fuel and moderator temperature coefficients at intermediate and later stages of burnup, low peaking factors and high delayed neutron fraction at the BOL. On the other hand, using a thickness 200 μm of SS-310 and FeCrAl with enrichment 5.06% and 5.22% respectively can achieve the same performance of Zirconium case (thickness 571 μm and enrichment 4.9%).
